# Smoking and Hypoalbuminemia as Risk Factors for Postoperative Complications in Reconstructive Hand and Forearm Surgery

**DOI:** 10.7759/cureus.99041

**Published:** 2025-12-12

**Authors:** Aneeq S Chaudhry, Alexander J Rodriguez, Andrew Liepshutz, Pura Rodriguez de la Vega, Rupa Seetharamaiah, Marcia Varella

**Affiliations:** 1 Department of Surgery, Florida International University, Herbert Wertheim College of Medicine, Miami, USA; 2 Department of Medical and Population Health Sciences Research, Florida International University, Herbert Wertheim College of Medicine, Miami, USA; 3 Department of Surgery, Baptist Hospital of Miami, Miami, USA

**Keywords:** hand surgery, hypoalbuminemia, orthopedic surgery, plastic surgery, ­reconstructive surgery, smoking

## Abstract

Background and objective

Limited evidence exists regarding the synergistic effects of smoking and hypoalbuminemia on postoperative complications in hand and forearm surgery. This study aimed to investigate the association between preoperative albumin levels, smoking status, and postoperative complications in US adults undergoing reconstructive procedures.

Methods

We conducted a retrospective cohort analysis of 20,725 adults identified in the 2011-2021 American College of Surgeons’ National Surgical Quality Improvement Program (ACS-NSQIP) database who underwent reconstructive hand and forearm procedures corresponding to 43 Current Procedural Terminology (CPT) codes. Smoking within one year of surgery and preoperative albumin levels below 3.5 g/dL were treated as primary independent variables. Binary logistic regression models adjusted for potential confounders were used to estimate associations.

Results

Among the patients, 21.1% experienced composite complications. Most were due to prolonged hospital stays lasting more than two days (17.9%), followed by readmissions (2.6%). After adjustment, smokers exhibited higher odds of complications compared to nonsmokers (odds ratio (OR): 1.16, 95% confidence interval (CI): 1.04-1.29). Hypoalbuminemia independently increased the odds of complications by 2.5 times (OR: 2.51, 95% CI: 2.25-2.80). No evidence of interaction between hypoalbuminemia and smoking was observed (p = 0.227).

Conclusions

Although no significant interaction effect was observed between smoking and hypoalbuminemia, these factors were independent risk factors for postoperative complications in reconstructive hand and forearm surgery. Our findings reaffirm the importance of considering and addressing these modifiable risk factors in surgical planning for this patient population to achieve optimal post-op outcomes.

## Introduction

Reconstructive hand surgery is becoming increasingly popular in the United States. According to the 2020 Plastic Surgery Statistics Report from the American Society of Plastic Surgeons, hand surgery ranked fifth among reconstructive procedures. The common operations include arthroplasty, tendon and nerve repair, skin grafts, and surgical debridement. In 2020, a total of 206,928 hand surgeries were performed, representing a 1% increase compared to 2019 [1].

Complications within 30 days of hand surgery occur in 2.5% of cases, with surgical site infection being the most common [2]. Despite the low incidence, such complications can greatly affect patients and healthcare resources, potentially leading to longer hospital stays, reoperations, higher infection risks, and other iatrogenic problems [2]. In addition to patient-level health impacts, complications also have negative economic implications for patients, hospitals, and healthcare systems. Estimated mean hospital costs for patients with complications ($36,060) were $19,626 (119%) higher than for those without complications ($16,434) [3]. Risk adjustment analyses have shown that the overall profit margin of healthcare institutions decreases from 5.8% for patients without complications to 0.1% for patients with complications [3]. For hand arthroplasty specifically, each complication was estimated to increase costs by an average of $1,076 [4].

Studies have evaluated risk factors associated with complications in reconstructive surgical procedures and findings suggest that history of comorbidities such as hypertension, diabetes, congestive heart failure, functional status, end stage renal disease, chronic obstructive pulmonary disease, anemia, as well as behavior factors such as smoking status, excessive alcohol consumption, sedentary habits, and smoking status were associated with increased risk of postoperative complications [5,6].

While several studies have analyzed the effects of cigarette smoking and hypoalbuminemia on common plastic surgical and orthopedic procedures, studies focused on surgical procedures involving the upper extremity are less common. With the increasing prevalence of elective surgical procedures involving the hand and forearm, we focused our attention on how common risk factors, smoking cigarettes and malnutrition, can impact the rate of postoperative complications. Moreover, the current literature does not adequately address the interaction between these two common risk factors. Given the similarities in the underlying pathophysiology of cigarette smoking and low serum albumin, potential synergistic effects are plausible. However, evidence remains limited regarding such combined effects and their influence on postoperative complications following hand and forearm surgery.

The objective of this study was to examine whether smoking and hypoalbuminemia are independently associated with postoperative complications following hand and forearm surgery in adults aged 18-90 years using data from a large national surgical database from 2011 to 2021. The primary outcome was a composite of postoperative complications, and the secondary objective was to assess for a potential interaction between smoking and hypoalbuminemia. We hypothesized that both smoking and hypoalbuminemia would be associated with increased odds of postoperative complications, and that their combined presence might further amplify this risk.

## Materials and methods

All analyses were based on deidentified data from the American College of Surgeons’ National Surgical Quality Improvement Program (ACS-NSQIP). The Institutional Review Board of Florida International University’s Herbert Wertheim College of Medicine determined that this study is exempt from review, as it does not meet the definition of human subjects research and involves only deidentified data.

Study design

We employed a retrospective cohort study using data from the ACS NSQIP database. The ACS NSQIP database gathers data from patients who undergo surgical procedures in both outpatient and inpatient settings through a direct review of their medical charts. This comprehensive data collection process spans the preoperative and intraoperative periods, as well as up to 30 days postoperatively.

Study population

Inclusion criteria required adult participants (ages 18-90) who had any of the relevant Current Procedural Terminology (CPT) codes recorded in the ACS NSQIP database from 2011 to 2021. Eligible procedures included tendon repair, arthroscopy/arthroplasty, peripheral nerve repair, drainage or foreign body removal, bone procedures, skin grafts, and debridement. A total of 43 CPT codes were included: 25000, 25320, 26356, 26370, 26410, 26418, 26426, 26442, 26460, 26500, 26502, 25332, 29844, 29846, 64713, 64831, 25040, 25076, 25111, 24666, 25035, 24363, 24587, 24615, 24685, 25400, 25405, 25440, 25545, 25574, 25607, 25608, 25609, 25628, 26735, 26746, 26765, 24105, 24201, 24341, 24342, 26615, and 26727 (Appendix 1).

For this analysis, forearm procedures were defined as those involving the distal portion of the upper extremity, including the forearm itself and extending proximally to the level of the elbow. Procedures involving the proximal arm or shoulder were not included. This definition ensured inclusion of reconstructive surgeries commonly classified as hand and forearm procedures in both orthopedic and plastic surgery practice.

Variables

The main independent variables were patient smoking status (a dichotomous variable, defined as cigarette use within one year of reconstructive hand surgery) and preoperative hypoalbuminemia, defined as a serum albumin level below 3.5 g/dL based on prior studies [17]. Additional covariates included age (measured in years), race (as recorded in the medical record), gender, and comorbidities. Comorbidities assessed were diabetes, hypertension, congestive heart failure (CHF), chronic obstructive pulmonary disease (COPD), dialysis, ventilator dependence, functional health status before surgery, disseminated cancer, immunosuppressive therapy, bleeding disorders, and systemic sepsis within 48 hours before surgery. All variables were obtained from the ACS NSQIP database.

Outcome measures

The main study outcome was a composite measure, defined as the occurrence of any selected major or minor postoperative complications within 30 days after surgery. Major complications include organ space infection, sepsis, septic shock, deep infection, wound dehiscence, pulmonary embolism, ventilator greater than 48 hours, reintubation, unplanned intubation, acute renal failure, cardiac arrest, myocardial infarction, stroke, coma greater than 24 hours, graft/prosthesis failure, peripheral injury, return to OR, unplanned reoperation, readmission, and length of hospital stay greater than two days. Length of stay greater than two days was included in the composite outcome as defined by NSQIP.

Although non-clinical factors can affect discharge timing, prior studies confirm that the procedures in our cohort are routinely performed as outpatient cases. Newman et al. reported a mean stay of 0.75 days for healthy patients undergoing distal radius fixation, with longer stays associated with increased complications [18]. Similarly, Donato et al. found that unplanned admissions or extended stays after outpatient hand surgery were uncommon and often linked to postoperative issues [19]. Minor complications include superficial surgical site infection, pneumonia, urinary tract infection, deep venous thrombosis, transfusion, and progressive renal failure. Secondary outcomes included the occurrence of all-cause mortality within 30 days of surgery, as well as the occurrence of major complications and minor complications assessed separately.

Statistical analysis

Stata software version 15.0 (StataCorp, College Station, TX) was used for all analyses. Descriptive statistics were used to summarize baseline characteristics. Bivariate analyses were performed using chi-square tests for categorical variables and t-tests for continuous variables. Multivariable logistic regression was used to examine the association between smoking, hypoalbuminemia, and 30-day postoperative complications, adjusting for demographic and clinical covariates. An interaction term between smoking and hypoalbuminemia was included to assess potential synergistic effects. Odds ratios (OR) with 95% confidence intervals (CI) were reported, and statistical significance was set at p<0.05.

## Results

A total of 105,488 patients from the ACS NSQIP were initially identified as having undergone reconstructive procedures of the hand and forearm. Of these patients, 57 were excluded due to missing complication data, and 84,742 were excluded for lacking records of preoperative albumin levels. This resulted in a final analytical sample of 20,725 patients, with 3661 (21.7%) having reported active smoking and 2037 (12.1%) having an albumin level below 3.5 g/dL (hypoalbuminemia).

The patient’s characteristics varied significantly by smoking status and albumin levels (Table 1). Smokers were younger, with a higher proportion aged 18-39 years (27.3% vs. 15.9%) and were more likely to be males (49.8% vs. 38%) compared to non-smokers. Additionally, the smokers group had a higher proportion of participants with a history of COPD than non-smokers (11.2% vs. 3.8%). Concerning albumin levels, those with low levels (<3.5 g/dL) were more likely to be older (39.5% aged ≥70), female (64.3%), have hypertension (58.2%), and have bleeding disorders (9.2%) compared to those with normal levels (≥3.5 g/dL).

**Table 1:** Baseline characteristics of adults who underwent hand and forearm surgery by smoking status and albumin levels ^*^Hematocrit levels are recorded as preoperative hematocrit levels. Low hematocrit was defined as a preoperative hematocrit below 41 for men and 36 for women BMI: body mass index; COPD: chronic obstructive pulmonary disease; CHF: congestive heart failure

Characteristics	Smoking status						Albumin (g/dL)				
	Non-smoker (n = 16,276)		Smoker (n = 4,449)			P-value	Normal (≥3.5, n = 18,288)		Low (<3.5, n = 2,437)		P-value
	N	%	N	%			N	%	N	%	
Age group, years					<0.001						<0.001
18-39	2580	15.9	1215	27.3			3552	19.4	243	10.0	
40-49	1960	12	801	18			2516	13.8	245	10.1	
50-59	3181	19.5	1163	26.1			3915	21.4	429	17.6	
60-69	3944	24.2	907	20.4			4294	23.5	557	22.9	
≥70	4611	28.3	363	8.2			4011	21.9	963	39.5	
Sex					<0.001						<0.001
Female	10,086	62	2235	50.2			10,755	58.8	1566	64.3	
Male	6190	38	2214	49.8			7533	35.7	871	35.7	
BMI, kg/m²					<0.001						<0.001
<18.5	296	1.9	144	3.3			354	2.0	86	3.7	
18.5-24.9	3981	25	1440	33			4717	26.3	704	29.9	
25-29.9	5071	31.8	1337	30.7			5717	31.9	691	29.3	
≥ 30	6578	41.3	1439	33			7143	39.8	874	37.1	
Race					<0.001						0.001
White	12,554	86.9	3328	84.1			14,010	86.2	1872	87.2	
Black	1211	8.4	485	12.3			1489	9.2	207	9.7	
Asian	481	3.3	55	1.4			502	3.1	34	1.6	
Other	198	1.4	88	2.2			253	1.6	33	1.5	
CHF					0.078						<0.001
No	16,112	99	4417	99.3			18,167	99.3	2362	96.9	
Yes	164	1	32	0.7			121	0.7	75	3.1	
COPD					<0.001						<0.001
No	15,655	96.2	3952	88.8			17,473	95.5	2134	87.6	
Yes	621	3.8	497	11.2			815	4.5	303	12.4	
Hypertension					<0.001						<0.001
No	8804	54.1	2808	63.1			10,592	57.9	1020	41.9	
Yes	7472	45.9	1641	36.9			7696	42.1	1417	58.2	
History of dialysis					0.107						<0.001
No	16,155	99.3	4426	99.5			18,205	99.6	2376	97.5	
Yes	121	0.7	23	0.5			83	0.5	61	2.5	
Cancer					0.023						<0.001
No	16,189	99.5	4437	99.7			18,220	99.6	2406	98.7	
Yes	87	0.5	12	0.3			68	0.4	31	1.3	
History of bleeding disorders					<0.001						<0.001
No	15,621	96	4328	97.3			17,737	97.0	2212	90.8	
Yes	655	4	121	2.7			551	3.0	225	9.2	
Preoperative systemic sepsis					<0.001						<0.001
No	15,893	97.7	4289	96.4			17,951	98.2	2231	91.6	
Yes	383	2.4	160	3.6			337	1.8	206	8.5	
History of diabetes					<0.001						<0.001
No	13,760	84.5	3921	88.1			15,757	86.2	1924	79.0	
Yes	2516	15.5	528	11.9			2531	13.8	513	21.1	
Functional health status					0.005						<0.001
Independent	15,570	96.9	4280	97.7			17,691	98.0	2159	90.2	
Dependent	499	3.1	101	2.3			364	2.0	236	9.9	
History of immunosuppressive therapy					<0.001						<0.001
No	15,490	95.2	4305	96.8			17,567	96.1	2228	91.4	
Yes	786	4.8	144	3.2			721	3.9	209	8.6	
Hematocrit^*^, %					0.699						<0.001
Normal	10,990	71.7	3051	72			13,161	76.6	880	36.8	
Low	4340	28.3	1187	28			4013	23.4	1514	63.2	

Complications occurred in 21.1% of the patient population who underwent hand and forearm surgery (Table 2). Overall, patients with preoperative systemic sepsis had the highest observed complication rate, with 78.5% experiencing a postoperative complication. Patients with hypoalbuminemia (<3.5 g/dL) had higher complication rates compared to those without hypoalbuminemia (50.1% vs. 17.2%). The complication rates in older patients were greater than the rates in younger patients. Higher complication rates were also found in those with CHF, COPD, hypertension, low hematocrit, decreased functional status, history of immunosuppressive therapy, history of bleeding disorders, and history of dialysis.

**Table 2:** Baseline characteristics of adults who underwent hand and forearm surgery by occurrence of any complications ^*^Hematocrit levels are recorded as preoperative hematocrit levels. Low hematocrit was defined as a preoperative hematocrit below 41 for men and 36 for women BMI: body mass index; COPD: chronic obstructive pulmonary disease; CHF: congestive heart failure

Characteristics	Occurrence of any complications				P-value
	No (n = 16357)		Yes (n = 4368)		
	N	%	N	%	
Smoking status					0.167
No	12,879	79.1	3397	20.9	
Yes	3478	78.2	971	21.8	
Albumin, g/dL					<0.001
≥3.5	15,141	82.8	3147	17.2	
<3.5	1216	49.9	1221	50.1	
Age group, years					<0.001
18-39	3264	86.0	531	14.0	
40-49	2340	84.8	421	15.3	
50-59	3600	82.9	744	17.1	
60-69	3928	81.0	923	19.0	
≥70	3225	64.8	1749	35.2	
Sex					0.016
Female	9655	78.4	2666	21.6	
Male	6702	79.8	1702	20.3	
BMI, kg/m²					<0.001
<18.5	298	67.7	142	32.3	
18.5-24.9	4186	77.2	1235	22.8	
25-29.9	5124	80.0	1284	20.0	
≥30	6516	81.3	1501	18.7	
Race					<0.001
White	12,551	79.0	3331	21.0	
Black	1401	82.6	295	17.4	
Asian	449	83.8	87	16.2	
Other	223	78.0	63	22.0	
CHF					<0.001
No	16,261	79.2	4268	20.8	
Yes	96	49.0	100	51.0	
COPD					<0.001
No	15,649	79.8	3958	20.2	
Yes	708	63.3	410	36.7	
Hypertension					<0.001
No	9574	82.5	2038	17.6	
Yes	6783	74.4	2330	25.6	
History of dialysis					<0.001
No	16,289	79.2	4292	20.9	
Yes	68	47.2	76	52.8	
Cancer					<0.001
No	16,298	79.0	4328	21.0	
Yes	59	59.6	40	40.4	
History of bleeding disorders					<0.001
No	15,960	80.0	3989	20.0	
Yes	397	51.2	379	48.8	
Preoperative systemic sepsis					<0.001
No	16,240	80.5	3942	19.5	
Yes	117	21.6	426	78.5	
History of diabetes					<0.001
No	14,097	79.7	3584	20.3	
Yes	2260	74.2	784	25.8	
Functional health status					<0.001
Independent	15,869	79.9	3981	20.1	
Dependent	265	44.2	335	55.8	
History of immunosuppressive therapy					<0.001
No	15,723	79.4	4072	20.6	
Yes	634	68.2	296	31.8	
Hematocrit^*^,%					<0.001
Normal	11,905	84.8	2136	15.2	
Low	3356	60.7	2171	39.3	

The overall composite complication rate included major complications, minor complications, and death (Table 3). Major complications occurred in 20.0% of patients, with the most frequent (17.9%) being extended hospital stay (length of hospital stay >2 days). The next most frequent major complications were readmission (2.7%) and return to the operating room (1.5%). Minor complications accounted for 2.2% of total complications. These included pneumonia, superficial surgical site infection, urinary tract infection, deep vein thrombosis/thrombophlebitis, and bleeding transfusions. The most frequent minor complications were superficial surgical site infection at 0.9% and bleeding transfusions at 0.5%. There were 52 deaths (0.25%) within 30 days postoperatively.

**Table 3:** Frequency of 30-day complications included as the main composite outcomes (NSQIP 2011-2021) ^*^Composite complications are total complications as denoted in Appendix 2 NSQIP: National Surgical Quality Improvement Program; CVA: cerebrovascular accident; CPR: cardiopulmonary resuscitation; DVT: deep vein thrombosis

	N	%
Death	52	0.25
Major complications	4195	20.0
Organ space infection	66	0.32
Sepsis	111	0.54
Septic shock	21	0.1
Deep surgical site infection	48	0.23
Wound dehiscence	48	0.23
Pulmonary embolism	20	0.1
Ventilator >48 hours	12	0.06
Unplanned intubation	22	0.11
CVA/stroke with neurological deficit	11	0.05
Return to the operating room	315	1.52
Myocardial infarction	19	0.09
Readmission	549	2.65
Cardiac arrest requiring CPR	20	0.1
Postoperative dialysis	2	0.01
Length of hospital stay >2 days	3714	17.92
Minor complications	463	2.23
Pneumonia	74	0.36
Superficial surgical site infection	191	0.92
Urinary tract infection	106	0.5
DVT/thrombophlebitis	18	0.09
Bleeding transfusions	102	0.49
Composite outcomes^*^	4368	21.08

In unadjusted models, hypoalbuminemia was associated with approximately a fivefold increase in the odds of complications (OR: 4.83, 95% CI: 4.42-5.28, p<0.001), while smoking status was not associated with complications (OR: 1.06, 95% CI: 0.98-1.15, p = 0.167) (Table 4). The association of smoking with post-surgical complications was as follows: OR: 1.12 (95% CI: 1.00-1.26, p = 0.052) after adjusting for age, sex, BMI, race, comorbidities, and the interaction between smoking and albumin. Hypoalbuminemia remained significantly associated with complications (OR: 2.42, 95% CI: 2.13-2.74, p<0.001), although the strength of association was attenuated. Because the interaction between smoking and hypoalbuminemia was not significant (p = 0.227), a second adjusted model without the interaction term was tested. In this model, smoking was significantly associated with post-surgical complications (OR: 1.16, 95% CI: 1.04-1.29, p = 0.007), and hypoalbuminemia was also significantly associated with the odds of complications (OR: 2.51, 95% CI: 2.25-2.80, p<0.001).

**Table 4:** Associations between smoking status, albumin levels and selected characteristics and the occurrence of post-op complications after hand and arm surgery ^*^Hematocrit levels are recorded as preoperative hematocrit levels. Low hematocrit was defined as a preoperative hematocrit below 41 for men and 36 for women OR: odds ratio; CI: confidence interval; BMI: body mass index; COPD: chronic obstructive pulmonary disease; CHF: congestive heart failure

Characteristics	Unadjusted model		Adjusted model 1 with interaction term		Adjusted model 2 without interaction term	
	OR (95% CI)	p-value	OR (95% CI)	p-value	OR (95% CI)	p-value
Smoking status						
No	Reference		Reference		Reference	
Yes	1.06 (0.98-1.15)	0.167	1.12 (1.00-1.26)	0.052	1.16 (1.04-1.29)	0.007
Albumin, g/dL						
≥3.5	Reference		Reference		Reference	
<3.5	4.83 (4.42-5.28)	<0.001	2.42 (2.13-2.74)	<0.001	2.51 (2.25-2.80)	<0.001
Smoking x Albumin <3.5 g/dL			1.17 (.91-1.49)	0.227	-	-
Age group, years						
18-39	Reference		Reference		Reference	
40-49	1.11 (0.96-1.27)	0.154	1.01 (0.85-1.19)	0.933	1.01 (.85-1.19)	0.909
50-59	1.27 (1.13-1.43)	<0.001	1.12 (0.96-1.30	0.15	1.12 (0.97-1.30)	0.134
60-69	1.44 (1.29-1.62)	<0.001	1.15 (0.99-1.34)	0.063	1.16 (1.00-1.34)	0.057
≥70	3.33 (2.99-3.72)	<0.001	2.11 (1.81-2.45)	<0.001	2.11 (1.81-2.46)	<0.001
Sex						
Female	1.09 (1.02-1.16)	0.016	1.03 (0.94-1.13)	0.536	1.03 (0.94-1.12)	0.541
Male	Reference		Reference		Reference	
BMI, kg/m²						
<18.5	1.62 (1.31-1.99)	<0.001	1.21 (0.94-1.57)	0.141	1.21 (0.94-1.57)	0.139
18.5-24.9	Reference		Reference		Reference	
25-29.9	0.85 (0.780-0.93)	<0.001	0.95 (0.86-1.06)	0.366	0.95 (0.86-1.06)	0.369
≥30	0.78 (0.72-0.85)	<0.001	0.92 (0.83-1.03)	0.143	0.92 (0.83-1.03)	0.14
Race						
White	Reference		Reference		Reference	
Black	0.79 (0.70-0.90)	0.001	0.80 (0.69-0.94)	0.005	0.80 (0.69-0.94)	0.005
Asian	0.73 (0.58-0.92)	0.008	0.71 (0.54-0.93	0.012	0.71 (0.54-0.93)	0.012
Other	1.06 (0.80-1.41)	0.664	1.16 (0.83-1.60)	0.368	1.16 (0.84-1.61)	0.361
CHF						
No	Reference		Reference		Reference	
Yes	3.97 (2.99-5.26)	<0.001	1.67 (1.17-2.37)	0.004	1.66 (1.17-2.37)	0.005
COPD						
No	Reference		Reference		Reference	
Yes	2.29 (2.02-2.60)	<0.001	1.28 (1.09-1.50)	0.003	1.28 (1.09-1.5)	0.003
Hypertension						
No	Reference		Reference		Reference	
Yes	1.61 (1.51-1.73)	<0.001	1.03 (0.94-1.14)	0.483	1.03 (0.94-1.13)	0.495
History of dialysis						
No	Reference		Reference		Reference	
Yes	4.24 (3.05-5.89)	<0.001	1.93 (1.29-2.88)	0.001	1.92 (1.28-2.87)	0.001
Disseminated cancer						
No	Reference		Reference		Reference	
Yes	2.55 (1.71-3.82)	<0.001	1.32 (0.82-2.13)	0.255	1.31 (0.81-2.11)	0.268
History of bleeding disorders						
No	Reference		Reference		Reference	
Yes	3.82 (3.30-4.42)	<0.001	2.12 (1.77-2.53)	<0.001	2.12 (1.78-2.53)	<0.001
Preoperative systemic sepsis						
No	Reference		Reference		Reference	
Yes	15.00 (12.19-18.46)	<0.001	11.98 (9.37-15.30)	<0.001	11.99 (9.38-15.31)	<0.001
History of diabetes						
No	Reference		Reference		Reference	
Yes	1.36 (1.25-1.49)	<0.001	1.02 (0.91-1.14)	0.756	1.02 (0.91-1.14)	0.76
Functional health status						
Independent	Reference		Reference		Reference	
Dependent	5.04 (4.27-5.94)	<0.001	2.21 (1.81-2.70)	<0.001	2.21 (1.80-2.70)	<0.001
Hematocrit^*^, %						
Normal	Reference		Reference		Reference	
Low	3.61 (3.36-3.87)	<0.001	2.49 (2.28-2.72)	<0.001	2.49 (2.28-2.72)	<0.001
History of immunosuppressive therapy						
No	Reference		Reference		Reference	
Yes	1.80 (1.56-2.08)	<0.001	1.15 (0.96-1.37)	0.142	1.14 (0.95-1.37)	0.148

## Discussion

Smoking influencing postoperative complications following hand and forearm surgery

Of interest, smoking has been shown to increase complications in a wide array of reconstructive plastic surgeries, including craniofacial and extremities, within the first 30 days of operation [7]. However, there is limited data on the link between smoking and complications associated with hand and forearm surgery. Nicotine, the primary chemical within cigarettes, has prothrombotic and vasoconstrictive effects due to increased platelet aggregation, microangiopathy, and macroangiopathy [8,9]. Additionally, nicotine decreases the proliferation of fibroblasts and macrophages that are essential for collagen formation and wound healing, while also altering the release of catecholamines essential for perfusion and epithelialization of the wound site [8]. These compromised molecular mechanisms are essential to the wound healing processes.

Consistent with the pathophysiology described above, a meta-analysis that utilized data from nearly 500,000 patients who underwent hip and knee arthroplasty, herniotomy, cholecystectomy, and colorectal resection across 140 cohort studies reported necrosis to be four times more frequent in smokers compared to non-smokers, whereas surgical site infection, dehiscence, and delayed healing to be two times more frequent in smokers [10].

Another study assessing 36,454 patients who underwent common plastic surgery procedures from 2005-2016 and contributing to the ACS-NSQIP database found that smokers had significantly increased deep incisional surgical-site infections, incisional dehiscence, and reoperation (p<0.01 for all) [11]. Yet, for superficial surgical site infections, there were no statistically significant differences between smokers and non-smokers. Another study assessing 40,465 patients who underwent a wide variety of plastic surgery procedures from 2007 to 2012 found that smokers who had undergone hand and upper extremity-specific procedures had increased surgical complications (OR: 2.21, 95% CI: 1.39-3.42, p<0.001) and increased wound complications (OR: 2.79, 95% CI: 1.68-4.52, p<0.001) [7].

The impact of cigarette smoking on hand and upper extremity surgery has also been evaluated [5,6]. Although the overall complication rate was low (2.54%), smokers experienced higher rates of postoperative complications within 30 days. Specifically, the ORs were: 1.10 (95% CI: 0.9-1.34) for minor complications, 1.30 (95% CI: 1.06-1.60) for major complications, 1.41 (95% CI: 1.16-1.72) for wound complications [5], 1.51 (95% CI: 1.22-1.85) for superficial site infections, 1.73 (95% CI: 1.34-2.22) for deep site infections, 1.50 (95% CI: 1.24-1.80) for reoperation, and 1.49 (95% CI: 1.23-1.80) for hospital readmission [6].

Contrary to the above results, one cohort study found no significant association between smoking and increased postoperative complications [12]. In this retrospective study, the data of patients who underwent either free flap transfer procedures or replantation/revascularization procedures of the upper extremity were analyzed from the ACS NSQIP. Of the 340 total patients, 70 underwent free flap transfer, and 270 underwent replantation and/or revascularization. The results yielded no association between cigarette smoking and 30-day postoperative complications following either type of upper extremity reconstructive procedure. Potential explanations for the differences in results were the scope of procedures performed, which related to digit microvascular and free flap transfers, not including procedures related to tendon repair and bone reconstruction in the hands, wrist, and forearm. It remains unclear whether surgeries in different regions of the upper extremity are differently affected by smoking, or if the observed differences are simply due to random variation, possibly related to smaller sample sizes.

Hypoalbuminemia and postoperative complications following hand and forearm surgery

Hypoalbuminemia has been previously found to be associated with post-surgical complications [13]. In retrospective cohort studies [14,15], patients undergoing surgery of the distal radius (open reduction and internal fixation) who were malnourished and for whom serum albumin was less than 3.5 g/dL had higher incidences of post-surgery complications when compared to patients with albumin levels within the reference range (OR: 4.8,; 95% CI: 2.47-9.66; p<0.05). Malnourished patients were also shown to have significantly longer hospital stays and an increased risk of 30-day postoperative morbidity and mortality [14], even after adjustment for patients’ history of diabetes, anemia, end-stage renal disease, chronic obstructive pulmonary disease, congestive heart failure, hypertension, BMI, and smoking status. Patients with hypoalbuminemia were found to have a higher risk of death (adjusted RR: 1.67, 95% CI: 1.07-2.62), development of minor complications (adjusted RR: 16.9, 95% CI: 5.40-52.68), and development of major complications (adjusted RR: 8.29, 95% CI: 5.20-13.21) [15]. Additionally, those with hypoalbuminemia showed increased rates of sepsis/septic shock, deep infection, ventilator requirement, and reintubation following hand surgery.

Potential for interaction between smoking and hypoalbuminemia on postoperative complications

Patients who had undergone cytoreductive surgery followed by hyperthermic intraperitoneal chemotherapy had the highest morbidity and mortality in those with lower serum albumin levels and positive smoking history (OR: 3.3, 95% CI: 1.5-7.5, p<0.003) [16]. Yet, the interaction between these factors was not assessed. Wilson et al. analyzed the effects of malnutrition and postoperative complications following distal radius fracture surgeries and found that smoking status did not affect albumin level availability [14].

Our findings highlight the strong association between hypoalbuminemia and postoperative complications following reconstructive hand and forearm surgery. The twofold increase in the odds of complications associated with hypoalbuminemia (<3.5 g/dL) reflects its role as a critical predictor of surgical risk. This result aligns with prior studies linking hypoalbuminemia to poor wound healing, increased infection rates, and prolonged hospital stays, likely due to its association with systemic inflammation and malnutrition. For instance, Luchetti et al. found that patients with hypoalbuminemia had a significantly increased risk of major complications (adjusted RR: 8.29, 95% CI: 5.20-13.21), minor complications (adjusted RR: 16.9, 95% CI: 5.40-52.68), and mortality (adjusted RR: 1.67, 95% CI: 1.07-2.62) after hand surgery. The twofold increase in the odds of complications associated with hypoalbuminemia (<3.5 g/dL) reflects its role as a critical predictor of surgical risk [15]. Similarly, Wilson et al. reported a fourfold increase in complications (OR: 4.88, 95% CI: 2.47-9.66) and a ninefold increase in mortality (OR: 9.23, 95% CI: 1.55-54.87) among orthopedic patients with albumin <3.5 g/dL [14].

Notably, length of stay >2 days was the most common major complication, reinforcing the clinical and economic burden associated with postoperative morbidity. Secondary outcomes, including readmissions, return to the operating room, and superficial surgical site infections, were more frequent in patients with complications. Adjusted analyses indicated an OR of 1.12 (95% CI: 1.00-1.26, p = 0.052) of complications also for patients who smoke. However, results showed a lack of significance for the interaction between smoking and hypoalbuminemia (p = 0.227), suggesting that these factors do not have a synergistic effect.

Of note, the small sample size of patients with both smoking exposure and low albumin levels limits the power to detect such interactions. Although the odds ratio for smoking was modest (OR: 1.16, p = 0.007) in the final adjusted model, even small increases in risk may be clinically meaningful at the population level, given the high prevalence of smoking. This finding supports the value of smoking cessation interventions as part of preoperative optimization, even for lower-risk procedures such as outpatient hand surgery. Nonetheless, larger studies are needed to further investigate this potential interaction.

This study contributes to the growing body of evidence on risk stratification in upper extremity surgery, highlighting hypoalbuminemia as a potentially modifiable risk factor. Preoperative nutritional interventions, including albumin supplementation and dietary optimization, may improve outcomes. Despite the limitations imposed by the exclusion of patients without albumin data, sensitivity analyses showed that demographic and comorbidity profiles were statistically comparable between patients with and without albumin data, based on our multivariable models adjusted for age, BMI, comorbidities, and smoking. Importantly, our results align with prior NSQIP analyses, such as the study by Luchetti et al, which employed generalized linear mixed modeling to account for procedural variability and likewise demonstrated that hypoalbuminemia is independently associated with increased complications after hand surgery [15]. Taken together, these findings underscore the consistency of our results within the broader literature on hypoalbuminemia as a surgical risk factor.

Limitations

A major limitation of this study relates to the large proportion of patients with missing data on preoperative albumin levels or smoking who were not analyzed. Of those patients excluded, 20.3% reported smoking and 10.5% reported an albumin level below 3.5 g/dl. Comparisons of characteristics in participants who were excluded demonstrated no apparent differences compared to the analytical sample, including no differences in the frequency of smoking status or the prevalence of hypoalbuminemia (Appendix 2, Table 6). However, the low percentage of patients for whom pre-op albumin levels records were available raises the possibility that albumin is not routinely measured preoperatively. A total of 84,742 patients (80.3%) were excluded for this reason, and patients without albumin measurements may differ systematically in their postoperative risk compared to those with recorded values.

While our analytical cohort remained robust in size, the absence of albumin data in the broader population may introduce selection bias and limit generalizability. Although the proportion of missing albumin values is considerable, it aligns with limitations reported in other national database studies. For example, Donato et al. also encountered a substantial amount of incomplete laboratory data in their analysis of outpatient hand surgery using the NSQIP [19]. Encouraging more consistent nutritional evaluation before surgery may strengthen future studies and improve perioperative risk stratification. As such, selection bias and concerns regarding the external validity of our findings cannot be excluded. Further studies using a sample for which key variables are systematically collected are still warranted.

Another limitation stems from the lack of details on key variables. For instance, the ACS-NSQIP data only provides self-reported information on whether patients have smoked within the past year. Characterization of smoking, such as cigarette dose (pack-years) and use of other nicotine-containing products, was not available. Such information is key to providing more precise and valid exposure data.

Lastly, the NSQIP database was not designed to systematically capture complications related more specifically to hand and forearm surgeries, such as prosthesis rejection or skin necrosis, nor did it include variables that could further confound the associations we report, such as chronic disease burden. Follow-up was limited to 30 days postoperatively. Therefore, the impact of the exposures assessed on long-term complications could not be evaluated. These limitations may have led to underestimation of complication rates and potential residual confounding. Despite these limitations, the NSQIP provided one of the largest nationwide cohorts of patients undergoing hand and forearm surgery, likely representative of the U.S. surgical population.

## Conclusions

In this exploratory analysis, hypoalbuminemia and smoking were both independently associated with postoperative complications in hand and forearm reconstructive surgery. Hypoalbuminemia was found to be a robust predictor of complications in hand and forearm reconstructive surgery, whereas smoking status showed a less pronounced effect. Future research should aim to further clarify the combined impact of smoking and hypoalbuminemia and refine predictive models for surgical risk assessment in larger longitudinal studies. Addressing nutritional deficiencies preoperatively is a practical strategy for enhancing patient outcomes.

## Appendices

Appendix 1

**Table 5:** List of Current Procedural Terminology (CPT) codes

CPT code	Description
25000	Incision, extensor tendon sheath, wrist (eg, deQuervains disease)
25320	Capsulorrhaphy or reconstruction, wrist, open (eg, capsulodesis, ligament repair, tendon transfer or graft) (includes synovectomy, capsulotomy and open reduction) for carpal instability
26356	Repair or advancement, flexor tendon, in zone 2 digital flexor tendon sheath (eg, no man's land); primary, without free graft, each tendon
26370	Repair or advancement of profundus tendon, with intact superficialis tendon; primary, each tendon
26410	Repair, extensor tendon, hand, primary or secondary; without free graft, each tendon
26418	Repair, extensor tendon, finger, primary or secondary; without free graft, each tendon
26426	Repair of extensor tendon, central slip, secondary (eg, boutonniere deformity); using local tissue(s), including lateral band(s), each finger
26442	Tenolysis, flexor tendon; palm AND finger, each tendon
26460	Tenotomy, extensor, hand or finger, open, each tendon
26500	Reconstruction of tendon pulley, each tendon; with local tissues (separate procedure)
26502	Reconstruction of tendon pulley, each tendon; with tendon or fascial graft (includes obtaining graft) (separate procedure)
25332	Arthroplasty, wrist, with or without interposition, with or without external or internal fixation
29844	Arthroscopy, wrist, surgical; synovectomy, partial
29846	Arthroscopy, wrist, surgical; excision and/or repair of triangular fibrocartilage and/or joint debridement
64713	Neuroplasty, major peripheral nerve, arm, open
64831	Suture of digital nerve, hand; 1 nerve
25040	Arthrotomy, radiocarpal or midcarpal joint, with exploration, drainage, or removal of foreign body
25076	Excision, tumor, soft tissue of forearm and/or wrist area, subfascial (eg, intramuscular); less than 3 cm
25111	Excision of ganglion, wrist (dorsal or volar); primary
24666	Open treatment of radial head or neck fracture, includes internal fixation or radial head excision, when performed; with radial head prosthetic replacement
25035	Incision, deep, bone cortex, forearm and/or wrist (eg, osteomyelitis or bone abscess)
24363	Arthroplasty, elbow (eg, total elbow)
24587	Open treatment of periarticular fracture and/or dislocation of the elbow; with implant arthroplasty
24615	Open treatment of acute or chronic elbow dislocation
24685	Open treatment of ulnar fracture, proximal end (eg, olecranon or coronoid process[es]), includes internal fixation, when performed
25400	Repair of nonunion or malunion, radius OR ulna; without graft (eg, compression technique)
25405	Repair of nonunion or malunion, radius OR ulna; with autograft (includes obtaining graft)
25440	Repair of nonunion, scaphoid carpal (navicular) bone, with or without radial styloidectomy (includes obtaining graft and necessary fixation)
25545	Open treatment of ulnar shaft fracture, includes internal fixation, when performed
25574	Open treatment of radial AND ulnar shaft fractures, with internal fixation, when performed; of radius OR ulna
25607	Open treatment of distal radial extra-articular fracture or epiphyseal separation, with internal fixation
25608	Open treatment of distal radial intra-articular fracture or epiphyseal separation; with internal fixation of 2 fragments
25609	Open treatment of distal radial intra-articular fracture or epiphyseal separation; with internal fixation of 3 or more fragments
25628	Open treatment of carpal scaphoid (navicular) fracture, includes internal fixation, when performed
26735	Open treatment of phalangeal shaft fracture, proximal or middle phalanx, finger or thumb, includes internal fixation, when performed, each
26746	Open treatment of articular fracture, involving metacarpophalangeal or interphalangeal joint, includes internal fixation, when performed, each
26765	Open treatment of distal phalangeal fracture, finger or thumb, includes internal fixation, when performed, each
24105	Excision, olecranon bursa
24201	Removal of foreign body, elbow area; deep (subfascial or intramuscular)
24341	Repair, tendon or muscle, elbow, each tendon or muscle, primary or secondary (excludes rotator cuff)
24342	Reinsertion of ruptured biceps or triceps tendon, distal, with or without tendon graft
26615	Open treatment of a single metacarpal fracture, meaning a surgical procedure to expose, realign, and stabilize a broken metacarpal bone in the hand, with or without internal fixation (like plates/screws) or external fixation
26727	Percutaneous skeletal fixation of an unstable phalangeal shaft fracture, specifically for the proximal or middle phalanx of the finger or thumb, involving manipulation to realign the bone fragments

Appendix 2 

**Table 6:** Distribution of baseline characteristics of adult patients who underwent reconstructive hand and forearm surgery ^*^Hematocrit levels are recorded as preoperative hematocrit levels. Low hematocrit was defined as a preoperative hematocrit below 41 for men and 36 for women BMI: body mass index; COPD: chronic obstructive pulmonary disease; CHF: congestive heart failure

Characteristics	Analytical sample (16,881)		Excluded (88,607)		P-value
	N	%	N	%	
Smoking status					<0.001
No	13,220	78.3	70,636	79.7	
Yes	3661	21.7	17,971	20.3	
Albumin, g/dL					0.006
≥3.5	14,844	87.9	3460	89.5	
<3.5	2037	12.1	405	10.5	
Total complications					<0.001
No	13,292	78.7	80,886	91.4	
Yes	3589	21.3	7664	8.7	
Age group, years					<0.001
18-39	2944	17.4	851	22.1	
40-49	2192	13	569	14.8	
50-59	3552	21	792	20.6	
60-69	4006	23.7	845	22.0	
≥70	4187	24.8	787	20.5	
Sex					<0.001
Female	10,143	60.1	2178	56.7	
Male	6738	39.9	1666	43.3	
BMI, kg/m²					0.527
<18.5	377	2.2	63	1.9	
18.5-24.9	4509	26.7	912	26.8	
25-29.9	5317	31.5	1091	32.0	
≥30	6678	39.6	1339	39.5	
Race					0.76
White	14,577	86.4	1305	85.9	
Black	1556	9.2	140	9.2	
Asian	485	2.9	51	3.4	
Other	263	1.6	23	1.5	
CHF					0.005
No	16,706	99	3823	99.5	
Yes	175	1	21	0.6	
COPD					<0.001
No	15,913	94.3	3694	96.1	
Yes	968	5.7	150	3.9	
History of hypertension					<0.001
No	9251	54.8	2361	61.4	
Yes	7630	45.2	1483	38.6	
History of dialysis					0.037
No	16,754	99.3	3827	99.6	
Yes	127	0.8	17	0.4	
History of any cancer					0.015
No	16,791	99.5	3835	99.8	
Yes	90	0.53	9	0.2	
History of bleeding disorders					<0.001
No	16,202	96	3747	97.5	
Yes	679	4	97	2.5	
Preoperative systemic sepsis					0.02
No	16,418	97.3	3764	97.9	
Yes	463	2.7	80	2.1	
History of diabetes					0.303
No	14,422	85.4	3259	84.8	
Yes	2459	14.6	585	15.2	
Pre-op functional health status					0.001
Independent	16,356	96.9	3494	97.9	
Dependent	525	3.1	75	2.1	
History of immunosuppressive therapy					0.052
No	16,101	95.4	3694	96.1	
Yes	780	4.6	150	3.9	
Hematocrit^*^, %					0.231
Normal	12,087	71.6	1954	72.3	
Low	4794	28.4	733	27.3	
